# Amplification and Manipulation of Nonlinear Electromagnetic Waves and Enhanced Nonreciprocity using Transmissive Space‐Time‐Coding Metasurface

**DOI:** 10.1002/advs.202105960

**Published:** 2022-02-10

**Authors:** Xin Wang, Jiaqi Han, Shuncheng Tian, Dexiao Xia, Long Li, Tie Jun Cui

**Affiliations:** ^1^ Key Laboratory of High‐Speed Circuit Design and EMC of Ministry of Education School of Electronic Engineering Xidian University Xi'an 710071 China; ^2^ Institute of Electromagnetic Space and the State Key Laboratory of Millimeter Waves Southeast University Nanjing 210096 China

**Keywords:** harmonic beam steering, nonlinearity, nonreciprocity, power amplifier, space‐time coding metasurface, transmission enhancement

## Abstract

A novel amplifier‐based transmissive space‐time‐coding metasurface is presented to realize strongly nonlinear controls of electromagnetic (EM) waves in both space and frequency domains, which can manipulate the propagation directions and adjust enhancements of nonlinear harmonic waves and break the Lorenz reciprocity due to the nonreciprocity of unilateral power amplifiers. By cascading the power amplifier between patches placed on two sides of the metasurface, the metasurface can transmit the spatial EM waves in the forward direction while blocking it in the backward direction. Two status of power amplifier biased at the standard working voltage and zero voltage are represented as codes “1” and “0,” respectively. By periodically setting adequate code sequences and proportions in the temporal dimension, according to the space‐time coding strategy, the amplitudes and phases of the harmonic transmission coefficients can be adjusted in a programmable way. A metasurface prototype is fabricated and measured in the microwave frequency to validate the concept and feasibility. The experimental results show good agreement with the theoretical predictions and numerical simulations. The proposed metasurface can achieve controllable harmonic power enhancements for flexibly configuring the power intensities in space, which enlarge and manipulate the quality of transmitting signals.

## Graphical abstract

Here the conceptual illustration of the proposed amplifier‐based transmissive space‐time coding metasurface is presented. According to the space‐time coding matrix, the nonreciprocal and enhanced nonlinear manipulation of electromagnetic waves can be obtained by switching the power amplifier integrated with the metasurface.

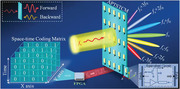

## 1 Introduction

During the past decades, metasurfaces have experienced a rich development as their distinctive properties for flexible electromagnetic (EM) wave manipulations. The metasurface is a 2D periodical structure consisting of sub‐wavelength inclusions in the ultrathin medium,^[^ ^1^, ^2^ ^]^ manipulating the phase, polarization, and amplitude of EM waves for the desired reflection, transmission, or scattering patterns. In contrast to metamaterials, metasurfaces are convenient to design, fabricate, and integrate with electronic components. Moreover, it has the advantage of lower losses, profile, and volume. A rich diversity of metasurface applications has been introduced over past decades, and it is hard to quote them all here. Some of the significant applications of metasurfaces are reported, such as polarization conversions,^[^ ^3^, ^4^, ^5^, ^6^ ^]^ absorptions,^[^ ^7^ ^]^ orbital angular momentum vortex waves,^[^ ^8^, ^9^, ^10^, ^11^ ^]^ holography,^[^ ^12^, ^13^, ^14^ ^]^ etc.

By evolving from the analog metasurface, the digital coding metasurface was first introduced by Cui et al. in 2014,^[^ ^15^ ^]^ which brings a new platform and opportunities for metasurface to take part in the digital world and acquired worldwide researcher's attention. To date, a variety of digital coding and programmable metasurfaces have been introduced to exhibit the rich versatility of engineering the EM wave in a programmable way, such as reprogrammable holograms,^[^ ^16^, ^17^ ^]^ independent amplitude and phase response control,^[^ ^18^ ^]^ light‐controlled programmable metasurface,^[^ ^19^ ^]^ space energy coding,^[^ ^20^ ^]^ digital message transmission,^[^ ^21^ ^]^ anomalously perfect reflection,^[^ ^22^ ^]^ programmable nonreciprocity,^[^ ^23^ ^]^ full space controls of EM wave,^[^ ^24^, ^25^ ^]^ polarization manipulation,^[^ ^26^, ^27^, ^28^ ^]^ millimeter‐wave imaging,^[^ ^29^ ^]^ reconfigurable beam scanning,^[^ ^30^ ^]^ and smart wireless power transmission.^[^ ^31^ ^]^ Digital coding metasurfaces usually integrate electronic‐controlled components such as positive‐intrinsic‐negative (PIN) diodes, varactors, and micro‐electro‐mechanical systems (MEMS). By digitally controlling these components, the quantized phase and magnitude response of the unit cell of the metasurface to the EM wave is achieved. For example, an n‐bit digital metasurface indicates the unit cell of metasurface has 2^n^ states of magnitude or phase response to the EM wave. The major challenge in the coding metasurface is to sophisticatedly manipulate the EM wave, which always requires more digital bit resources for the higher resolution of phase or magnitude response. Multiple‐bit metasurface generally combines with a complicated bias circuit, structure, and control system. Higher digital bits programmable metasurface could also be achieved by employing the varactors, always accompanied by higher losses.

More recently, amplifier‐based metasurfaces have aroused more and more interest from researchers due to their extraordinary physical characteristics, such as magnetless nonreciprocity,^[^ ^32^, ^33^, ^34^ ^]^ controlling reflection enhancement to incident EM waves,^[^ ^35^ ^]^ polarization selection,^[^ ^36^ ^]^ magnetless gyrotropic response to EM waves,^[^ ^37^, ^38^ ^]^ high power absorption,^[^ ^39^ ^]^ space energy coding,^[^ ^20^ ^]^ full‐duplex reflective beam steering,^[^ ^40^ ^]^ spatial‐wave multiplication,^[^ ^41^ ^]^ and prisms‐like nonreciprocal EM wave refraction.^[^ ^42^ ^]^ Another advanced nonlinear manipulation of EM waves via transistor‐based metasurfaces has been reported.^[^ ^43^, ^44^, ^45^ ^]^ Combining metasurface with amplifiers could bring new physical characteristics, nonreciprocity, which has the advantage of lower volume compared with conventional magnet devices. Moreover, this makes it possible to integrate with semiconductor technology, appropriate in the microwave and millimeter‐wave regime. However, the current configuration of amplified‐based metasurfaces is only fixed or programmable spatial responses to EM waves and does not exploit the time domains.

Space‐time coding metasurface^[^ ^46^ ^]^ has attracted widespread attention from scientists and engineers because it can manipulate EM waves in the space and time domain. Space‐time coding metasurface exhibits some entirely new physical phenomena and potential applications, such as linear and nonlinear polarization syntheses,^[^ ^47^ ^]^ magnetless nonreciprocity,^[^ ^48^, ^49^ ^]^ Doppler cloaks,^[^ ^50^ ^]^ and harmonics engineering.^[^ ^46^, ^51^, ^52^, ^53^, ^54^ ^]^ Moreover, the space‐time coding metasurface could be achieved by using the graphene material to manipulate the harmonics waves at THz frequency.^[^ ^55^ ^]^ Besides that, space‐time coding metasurface put forward a new framework of the wireless communication system by integrating the function of baseband calculation, leading to a significant simplification of the architecture of the wireless communication system.^[^ ^56^, ^57^, ^58^, ^59^, ^60^ ^]^ It is worth noting that the space‐time coding metasurfaces, as mentioned above, are all based on the utilization of devices such as PIN, MEMS, and varactors which are all losses devices at microwave or THz band. On the other hand, the effective working distance is critical to any wireless device, which determines the total system's efficiency, coverage, and cost. There are two ways to enlarge the coverage of wireless communication or remote system: one is to enhance the intensity of the output signal to improve the transmission coverage by adding a power amplifier module; the other is to reduce the noise of receiving system to increase the receiving scope via low noise amplifier.

In this paper, we present a novel amplifier‐based transmissive space‐time coding metasurface (APTSTCM), which exploits the nonreciprocal nonlinear amplifier‐based metasurface in the temporal domains. In contrast to space‐time coding metasurface integrated with other lossy components, APTSTCM could enhance the propagation of EM waves while inheriting the capabilities of harmonics beam engineering. Furthermore, it has nonreciprocal propagation properties in terms of the unilateral amplification of the power amplifier. The harmonic phase control and enhancement adjustment can be achieved readily by periodically switching amplifiers according to the space‐time coding strategy controlled by field‐programmable gate array (FPGA). Besides that, we can also adjust the period of time modulation to control the harmonic frequencies independently. Moreover, quantizing the time with a higher digital bit number could obtain the sophisticated manipulation of the outgoing harmonic wave, corresponding to manipulating EM waves with the higher bit coding digital metasurface and simplifying the metasurface architecture.

The proportion of harmonic components to the fundamental component usually decides the nonlinearity of systems and devices. One of the most critical characteristics of the space‐time coding metasurface is to manipulate the outgoing harmonic waves in both space and frequency domains. In this regard, the harmonics proportion (or nonlinearity) enhancement is prominent to the space‐time coding metasurface because it could increase the harmonic‐wave coverage and improve the signal quality. The proposed approach extends the scope of the space‐time coding metasurface, which has potential applications in the new wireless communication system, simultaneously wireless power and information delivery, wireless repeater, and radar system. Nonreciprocity is now a significant scientific and technological branch from microwave to optical frequencies. The proposed metasurface could enhance the incident wave along the forward direction and block it from the opposite direction. In addition, the architecture of the proposed approach could generate harmonic waves along the forward direction. Thus, the proposed metasurface could be a considerable method to realize novel applications in one‐way screens. High isolation of the proposed metasurface represents notable nonreciprocity, which could be an extraordinary solution to the potential application of spatial isolators.

## 2 Results

### 2.1 Basic Theory of APTSTCM

#### 2.1.1 Radiation Pattern Synthesizing

**Figure 1:**
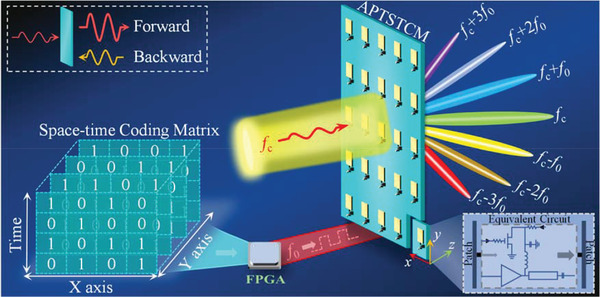
Conceptual illustration of the proposed amplifier‐based transmissive space‐time‐coding metasurface. The arbitral manipulation of EM waves in both frequency and space domain is obtained by modulating the status of the power amplifier integrated with the metasurface, according to the space‐time coding matrix. The nonreciprocity is achieved by enlarging the forward incident wave and blocking it from the backward of the prototype.

We assume that a 1‐bit APTSTCM consists of a 2D array of M×N cells loaded with amplification circuits, as shown in **Figure** **1**. The proposed prototype can manipulate the linearly *y*‐polarized wave because the designed patch placed on two sides of the manufactured metasurface merely works in the linear polarization mode. The outgoing harmonics waves keep the same polarization as the incident wave. By switching the amplifier, the transmission coefficient of the unit cell at different harmonics can be dynamically controlled with the desired phase or amplitude. The Digital code bit represents the status of the transmission coefficient. For instance, 1‐bit indicates the two different transmission coefficients. Each unit cell's transmission coefficient is periodically altered, determined by the space‐time coding matrix, as depicted in the bottom‐left corner of Figure 1. We assume the matrix entries as the location of the unit cell in the array. For simply illustrating the relationship of the matrix element and time modulation, the eight‐bit code of the matrix element represents the code sequence in the time domain. Therefore, the space‐time coding matrix method makes it possible to be numerically controlled by the digital system. If the period of modulation is much greater than the incident radio frequency wave period and by neglecting the coupling of each cell in the array, the radiation pattern with incidence wave *e ^j*ω*^* c*^t^* may be written as

(1) $$\begin{array}{ccc} S\left(\theta ,\varphi ,z,t\right) & = & \sum_{n=1}^{N}\sum_{m=1}^{M}E_{mn}\left(\theta ,\varphi \right)T_{mn}\left(z,t\right)\exp\left\{-jk\right.\left[\left(m-1\right)\right.\\ & & \left.\times \left.d_{x}\sin\theta \cos\varphi +\left(n-1\right)d_{y}\sin\theta \sin\varphi \right]\right\} \end{array}$$

where *E_mn_* (*θ,φ*) and *k* is the (*m*, *n*)th cell factor of the outgoing wave at frequency *f*_c_, *θ* and *φ* are the elevation and azimuth angles, respectively. *d_x_* and *d_y_* are the period distance of cells along the horizontal and vertical axes. Based on time modulated array theory,^[^ ^61^ ^]^ *T_mn_* (*z*,*t*) is a periodical function of time as transmission coefficient of the (*m*, *n*)th unit cell, which is defined as a linear combination of shifted pulse functions over one period

(2) $$T_{mn}\left(z,t\right)=\sum_{l=1}^{L}T_{mn}^{l}P_{mn}^{l}\left(t\right)u\left(z\right)\begin{array}{cc} & \left(0<t\leq T_{0}\right) \end{array}$$

As indicating the nonreciprocity of APTSTCM, the Heaviside step function *u*(*z*) is applied in the transmission function. *L* is an integer defined as the length of the time coding sequence. *T^l^_mn_* = *A^l^_mn_* exp(*jϕl*), is the transmission coefficient of (*m*, *n*)th element in the *l*th time‐coding series. And the pulse function *P^l^_mn_* (*t*) is given by

(3) $$P_{mn}^{l}\left(t\right)=\left\{\begin{array}{cc} 1, & \left(l-1\right)\sigma \leq t\leq l\sigma \\ 0, & 0<t<\left(l-1\right)\sigma ,l\sigma <t<T_{0} \end{array}\right.$$

where *σ *= *T*_0_ */L* is the pulse width. It is worth noting that the transmission coefficient is a periodical function of time along the positive *z*‐axis direction. Thus, we can rewrite the *T_mn_* (*z*,*t*) into Fourier series in the region *z* > 0 as

(4) $$T_{mn}\left(t\right)=\sum_{p=-\infty }^{\infty }c_{{}_{p}}^{mn}\exp\left(jp\omega_{0}t\right)$$

The above *ω*_0_ *= *2*π/Τ*_0_ is the angular frequency of time modulation. According to Fourier transform theory, the coefficient is given by

(5) $$\begin{array}{cc} c_{{}_{p}}^{\mathrm{mn}}= & \frac{1}{T_{0}}\int_{0}^{T_{0}}T_{\mathrm{mn}}\exp\left(-\mathrm{jp}\omega_{0}t\right)dt\\ = & \sum_{l=1}^{L}\frac{1}{L}T_{\mathrm{mn}}^{l}\sin c\left(\frac{p\omega_{0}\sigma }{2}\right)\exp\left(-j\frac{2l-1}{2}p\omega_{0}\sigma \right) \end{array}$$

Then the far‐field pattern at the positive *p*th harmonic frequency (*f*_c_ + *f_p_*) can be approximately expressed as

(6) $$\begin{array}{ccc} S\left(\theta ,\varphi ,z,t\right) & = & \sum_{n=1}^{N}\sum_{m=1}^{M}c_{p}^{mn}E_{mn}\left(\theta ,\varphi \right)\exp\left\{-jk\left[\left(m-1\right)d_{x}\sin\theta \cos\varphi \right.\right.\\ & & \left.\left.+\left(n-1\right)d_{y}\sin\theta \sin\varphi \right]\right\} \end{array}$$

According to this approximate formula, without considering the diffraction of the incident wave and ignoring the coupling of adjacent cells of the metasurface, harmonic power distribution in the far‐field space can be synthesized by Equation (6). In contrast, the fundamental frequency beam is pointed in the fixed direction because the phase response of the transmission coefficient is constant. On the other hand, arbitrarily manipulating the radiation pattern of harmonics is achieved by adjusting the space‐time coding matrix.

**Figure 2:**
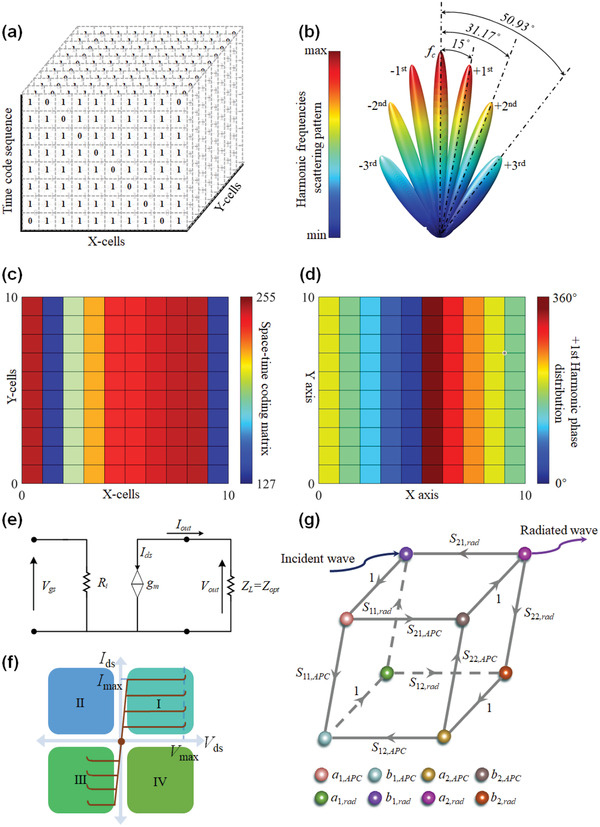
APTSTCM synthesis and analysis. a) The 10 × 10 × 8 dimensional space‐time coding matrix. b) Harmonic frequencies beam radiation patterns. c) Decimalized space‐time coding matrix. d) Phase distribution of +1st harmonic beam on metasurface. e) Theoretical power amplifier model. f) *I*–*V* region of the theoretical power amplifier. g) Scattering parameters model of APTSTCM element.

The time‐coding strategy is crucial for APTSTCM to manipulate the harmonics scattering patterns, including harmonic frequency control, harmonic beam scanning, harmonic power intensity adjusting, and wavefront engineering. In the 1‐bit case, for simplicity, the transmission coefficient has two values of 0 and 1 according to code “0” and “1”, respectively. Thus, the far‐field pattern of each harmonic can be synthesized via the 3D space‐time coding matrix defined by the spatial location of the unit cell of APTSTCM and the coding sequence in the time domain. From **Figure** **2**a, a space‐time coding matrix with (10,10,8) dimensions is considered here as an example to illustrate the synthesis method. Assuming that the adjacent units are spaced at half a wavelength of the incident wave, harmonic frequencies radiation patterns in the *xz*‐plane are depicted in Figure 2b. The value of the space‐time coding matrix is a decimalized 8‐bit hex number ranging from 127 to 256, considering that there is only one “0” in the 8‐bit hex code, as shown in Figure 2c. Furthermore, based on the theory of APTSTCM, the phase distribution on the metasurface of the +1st harmonic beam is shown in Figure 2d. We can observe that harmonics power distributions at the far‐field are symmetrical along with the radiation pattern of the fundamental harmonic scattering pattern, as shown in Figure 2b. It should be noted that the case mentioned above is assumed with the normalized transmission coefficient to explain the APTSTCM theory for simplicity. In most general situations, the magnitude of the transmission coefficient indicates whether the device is lossy. So the appropriate model of transmission coefficient is essential to APTSTCM.

#### 2.1.2 Transmission Coefficient Modeling

In contrast to other components (PIN diodes, MEMS, varactors) integrated metasurface, amplifier‐based metasurface^[^ ^35^, ^62^ ^]^ could amplify the incident wave rather than attenuate it. In the higher frequency range, this is an exceptionally prominent advantage for enhancing the coverage of metasurface, improving the total system efficiency, reducing the device's volume, and boosting the signal quality. The transmission coefficient of APTSTCM can be divided into two parts, the factor of the patches and the transmission factor of the amplifier. The characteristics of the patches are proportional to the designed passive structure, which is constant. Interestingly, the transmission factor of the amplifier can be adjusted by the bias voltage, providing the ability to manipulate the transmission coefficient arbitrarily. A theoretical model of an amplifier is considered here, a voltage‐controlled current source model, as shown in Figure 2e. Of great importance is the *I*–*V* region of the amplifier depicted in Figure 2f, which demonstrates the fundamental principle of optimum output power impedance *Z*_opt_ = 2*V*_dc_/*I*_max_ based on load‐pull theory.^[^ ^63^ ^]^ The amplifier is a nonreciprocal device^[^ ^64^, ^65^ ^]^ because the amplifier is just operated in I and III in the *I*–*V* region. Assuming *Z*_L_ = *Z*_opt_ and the transmission coefficient identical with the gain of an amplifier in the CLASS A status may be written as

(7) $$G=\frac{g_{m}^{2}R_{i}V_{\max}}{I_{\max}}$$

where *g_m_* is the transconductance and *R_i_* is the amplifier's input resistance. Here, we can see that the gain of an amplifier in the linear region is a constant. If we adjust the bias of gate voltage to control the threshold of an amplifier, the gain of an amplifier with settled input power is modified as

(8) $$G\left(\phi \right)=\frac{g_{m}^{2}V_{\max}R_{i}}{\pi^{2}I_{\max}}{\left(\sin\phi +\frac{\phi }{2}\right)}^{2}$$

where *ϕ* is the conduction angle of the amplifier controlled by the bias voltage. This relation extends the freedom of adjusting the gain of an amplifier. However, it needs to pay attention to handling the bias control voltage because it could impact the amplifier's nonlinearity. On the other hand, the gain could also be changed by periodically switching the amplifier. The temporal modulation gain of the amplifier is given by

(9) $$G\left(\phi ,t\right)=G\left(\phi \right)C\left(l\right)P^{l}\left(t\right)$$

Equation (9) denotes that the temporal modulation function could modify the gain of the amplifier *P^l^* (*t*) according to the *l*th code of the space‐time coding sequence. Furthermore, a period function could be expressed in the Fourier series. Thus, the temporal modulation gain at harmonic frequencies is modified by

(10) $$\begin{array}{ccc} G(\phi ,t) & = & \sum_{p=-\infty }^{\infty }\sum_{l=1}^{L}\frac{1}{L}G\left(\phi \right)C\left(l\right)\sin c\left(\frac{p\omega_{0}\sigma }{2}\right)\exp\left(-j\frac{2l-1}{2}p\omega_{0}\sigma \right)\\ & & \exp\left[j\left(\omega_{c}+p\omega_{0}\right)t\right] \end{array}$$

By combing the factor of patches with the gain of the amplifier, with the assumption that two patches are well‐matched, then the transmission coefficient of elements may be written as

(11) $$T\left(\phi ,t\right)=\eta_{r}\eta_{t}G\left(\phi ,t\right)$$

where *η*_r_ and *η*_t_ is the receiving and transmitting factor of two patches placed on two sides of the metasurface, respectively. Integrating the amplifier and patch has excellent advantages for reducing the device's size, enhancing coverage, increasing the system's efficiency, nonreciprocal transmission, and improving the transmissive signal quality. Besides that, the strong nonlinearity of the power amplifier is appropriate to space‐time coding metasurface, which could enhance the power intensity of harmonic distribution in space. However, the most prominent challenge of amplifier‐integrated metasurface is stability, which determines whether the devices can work smoothly and harmlessly in complex scenarios.

#### 2.1.3 Stability of APTSTCM

For any power amplifier metasurface, the most significant challenge is stability, which is mainly dependent on the feedback power from output to the input of the amplifier.^[^ ^66^ ^]^ The element of amplifier‐integrated metasurface usually consists of an amplifier circuit (APC) and passive structures. Therefore, the power amplifier can strengthen the signal from the receiving patch to the transmissive patch.^[^ ^34^, ^35^ ^]^ The coupling factor of the patch is key to the stability of the APTSTCM, which creates a feedback path to the amplifier assembled on the metasurface. Therefore, minimizing the feedback is a much more challenging task in an amplifier‐based metasurface. Similar to ref. [61], to indicate the stability of APTSTCM, a scattering parameters model is constructed as Figure 2g shows. Based on the Mason rules, the reflection coefficient for both input and output of this device may be expressed as

(12) $$\Gamma_{\mathrm{IN}}=\frac{S_{11,\mathrm{APC}}\left(1-S_{22,\mathrm{APC}}S_{22,\mathrm{rad}}\right)+S_{21,\mathrm{APC}}S_{12,\mathrm{APC}}S_{22,\mathrm{rad}}}{\Delta }$$

(13) $$\Gamma_{\mathrm{OUT}}=\frac{S_{22,\mathrm{APC}}\left(1-S_{11,\mathrm{rad}}S_{11,\mathrm{APC}}\right)+S_{21,\mathrm{APC}}S_{12,\mathrm{APC}}S_{11,\mathrm{rad}}}{\Delta }$$

where

(14) $$\begin{array}{cc} \Delta = & 1-S_{11,APC}S_{11,rad}-S_{22,APC}S_{22,rad}-S_{21,APC}S_{12,rad}\\ & -S_{11,rad}S_{22,rad}S_{12,APC}S_{21,APC}-S_{11,APC}S_{22,APC}S_{12,rad}S_{21,rad}\\ & +S_{12,rad}S_{21,rad}S_{12,APC}S_{21,APC}+S_{11,APC}S_{11,rad}S_{22,APC}S_{22,rad} \end{array}$$

Equations (12) and (13) denote the total reflection coefficient of the amplifier‐based metasurface, considering the *S* parameter of amplifier circuit (APC) and patches. The reflection coefficients need to fulfill the conditions |*Γ*_IN_| < 1, |*Γ*_OUT_| < 1 to guarantee that the device is in the unconditional stable region. It is worth noting that the isolation from output to input for reflective and transmissive power amplifying metasurface is a critical factor in the device's stability. Recently, several methods have been reported to improve amplifier‐based metasurfaces’ stability, such as polarization isolation^[^ ^61^ ^]^ and space isolation.^[^ ^34^ ^]^ Therefore, a rigorous design of APTSTCM is required to ensure the reliability of the device.

### 2.2 Method and Materials

In order to validate the aforementioned concepts of APTSTCM, a 1‐bit APTSTCM consisting of 5 × 5 elements is designed and fabricated for enhancing and engineering the harmonic power distribution. The 1‐bit APTSTCM element consists of five parts: receiving patch, transmissive patch, phase compensation line, amplifier circuit, and amplifier bias control circuit. A multilayer printed circuit board (PCB) technology is applied for combining these five parts. Furthermore, reasonable design steps are required to guarantee the desired outcomes.

1) Designing the patch dimension for receiving and transmitting EM waves.
2) Developing amplifier and bias control circuit for temporal digitally coding realization.
3) Calibrating the length of phase compensation line in terms of the incident wave phase distribution on the plane of receiving side of the metasurface.
4) Combining receiving and transmitting patches, phase compensation line, amplifier, and bias control circuit into an element to realize the time coding element.
5) Merging M×N elements to form a 1‐bit APTSTCM.

The APTSTCM element can be divided into three parts: the patch, coding amplification circuit, and phase compensation line. One of the most critical matters for patch design is easy to integrate with an amplifier circuit. Additionally, the performance of the patch needs to be considered carefully. Besides that, the interface voltage of the amplifier and FPGA I/O port controlled by the bias control circuit should also be taken into account.

#### 2.2.1 The Performance of Passive Patch

**Figure 3:**
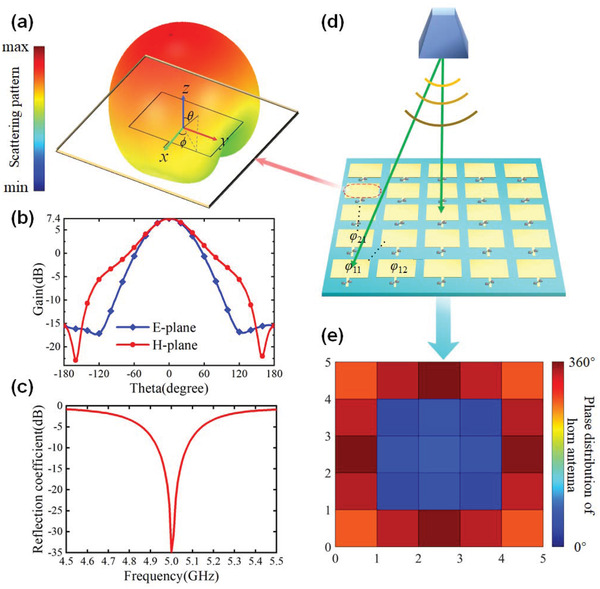
Radiator performance and phase distribution on metasurface. a) 3D view of the patch element and radiation pattern. b) Gain of the patch element at 5 GHz. c) Reflection coefficient of the patch element. d) Scheme of the horn antenna illuminating on the proposed metasurface. e) Phase distribution of horn antenna on the metasurface.

A classical rectangular metallic patch is chosen as the radiator for receiving and transmitting the incident wave, as shown in **Figure** **3**a. The metallic patch is located on the substrate with thickness *h*_1_ = 1.524 mm, relative dielectric constant *ε_r_* = 3.7, loss tangent tan *δ* = 0.004, and another side of the substrate is the ground plane. The exact sizes of the metallic patch element are *L*_a_ = 14.5 mm, *W*_a_ = 25 mm, *L*_z _= 4.05 mm, *W*_z _= 0.6 mm. By using commercial full‐wave simulation software, ANSYS Electromagnetics Suite, to analyze patch performance, more than 7.2 dBi gain at the boresight of the patch could be achieved, as depicted in Figure 3b. It can be seen from the reflection coefficient shown in Figure 3c that this patch resonates at 5 GHz.

#### 2.2.2 Phase Compensation Line Calculation

The illuminated wave to the metasurface from the feeding horn is a sphere rather than a plane wave, as depicted in Figure 3d. So the distribution of phase and magnitude of incident wave on each element of the metasurface is different. Therefore, compensating for the phase difference of the impinged wave on the metasurface is significant to accurate beam steering. There are two methods to balance the phase difference from the feeding horn antenna. One is to insert a phase compensation line between receiving and transmitting patches. The other is coding the phase difference in the space‐time code matrix for compensation, leading to a more complex coding matrix. For observing the results of the space‐time coding strategy more intuitively, the first method is preferred as the phase compensation manner. The phase distribution *φ_mn_* of each metasurface element is depicted in Figure 3e. Moreover, it is easy to calculate half of the phase compensation line by utilizing the relation *L_mn_* = *λ*_c_ *φ_mn_*/720. It should be noted here that the characteristic impedance of the phase compensation line is *Z*_0_ = 50 Ω.

#### 2.2.3 Amplifier and Bias Control Circuit

The power amplifier and its bias control circuit are essential in realizing the APTSTCM for harmonic generation, scanning, and enhancement. A good gain and isolation of the amplifier are well‐considered to ensure the stability of APTSTCM. Hence, the commercial amplifier GALI‐1+ manufactured by Mini‐Circuits is applied for the amplification circuit. This amplifier has already been internally matched to 50 Ω, which is convenient to be integrated with the patch. Good gain and isolation guarantee the stability of this APTSTCM. The low noise figure of this amplifier minimizes the noise while amplifying the incident wave received from the patch.

**Figure 4:**
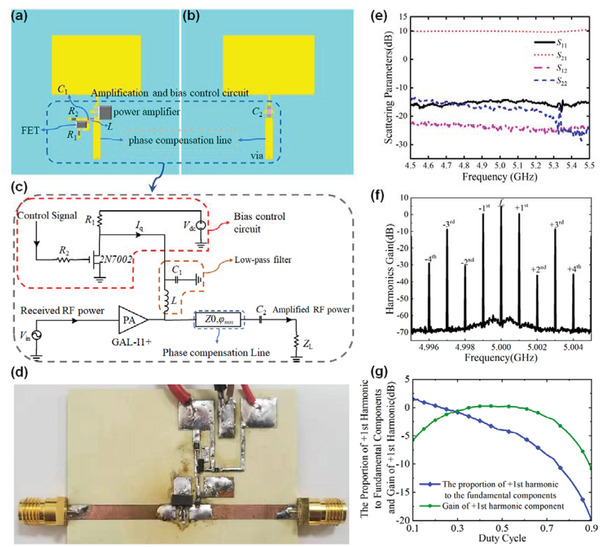
Performance of amplifier and bias control circuit, including harmonic frequencies generation and S parameters. a,b) Amplification and control circuit placed on the top and bottom of APTSTCM unit cell. c) Schematic of the amplification and bias control circuit. d) Fabricated amplifier circuit. e) Measured results of scattering parameters of the fabricated amplifier circuit. f) Harmonic power distribution in the spectrum of the amplification circuit. g) Measured results of the nonlinearity of power amplifier changing with the duty cycle in the modulation frequency 1 MHz, in which the central frequency is 5 GHz.

**Figure** **4**a,b shows that the amplification and bias control circuits are cascaded between the receiving and transmissive patches. For simply illustrating the principle of applied circuits, Figure 4c shows the schematic of the proposed circuits. A low‐pass filter consists of an inductor *L* (10 nH) and a capacitor *C*_1_ (100 pF) that can block the amplified signal from the output terminal of the amplifier to the bias control circuit while enabling the control signal to pass through. For isolating the DC voltage to the patch, a capacitor *C*_2_ (4.7 pF) is inserted between the phase compensation line and the transmissive patch. On the other hand, with consideration of digitally controlling the power amplifier, the n‐channel enhancement mode field‐effect transistor (FET) 2N7002 manufactured by Nxperia is utilized as a critical component to the bias control circuit. Two resistors, *R*_1_ (40 Ω) and *R*_2_ (1000 Ω), are implemented to limit the current from the DC power supplier and FPGA I/O port in case of damage to the FET and FPGA.

Thus, the resistance of channel *R*_ds_ from drain to source of FET can be adjusted by the output voltage from the FPGA I/O port. For the high output voltage of FPGA coded as “0,” *R*_ds_ of FET becomes near zero, leading to the voltage to amplifier bias port being zero. In the low output voltage of FPGA coded as “1,” FET is effectively turned off owing to that *R*_ds_ becomes much large at the time, and the voltage to the amplifier is *V*_d_ = *V*_dc_ − *I*_q_ *R*_1_. From Figure 4d, the amplification circuit and bias control circuit are fabricated to validate the feasibility of the temporal modulation of an amplifier. Measured results show good performance of amplification circuit demo as depicted in Figure 4e. By periodical switching amplifier via the bias control circuit, the harmonics could be excited with the settled input continuous‐wave (*f*_c_ = 5 GHz), as shown in Figure 4f, which indicates the strong nonlinearity of the amplifier. Considering the frequency (*f*_0_ = 1 MHz) and the duty cycle (50%) of time modulation, the intensity of even harmonic frequencies is much lower than others. Since the modulated wave to the power amplifier is a square wave, each harmonic frequency intensity could be adjusted simultaneously. Considering the duty cycle 0.5 (50%) of time modulation and the central frequency 5 GHz, the even coefficients of Fourier transform of the modulated square wave is zero: $\int_{0}^{T_{0}/2}\exp\left(-j2n\omega_{0}t\right)dt\;=\;0,n\in Z\;\mathrm{and}\;n\neq 0$, denoting that no even harmonic frequencies (*f*_c_+*f*_2_ *_n_*) exist theoretically. From Figure 4f, the gain of the even harmonic components (*f*_c_+*f*_2_ *_n_*) is at least −30 dB lower than that of the fundamental, which shows good agreement with the theoretical prediction.

The proposed metasurface's nonlinearity is mainly due to the nonlinearity of the power amplifier integrated with the unit cell. To effectively illustrate the power amplifier's nonlinearity, we measured the proportion of the first positive harmonic component to the fundamental component of the amplifier changing with the duty cycle, in which the modulation frequency is 1 MHz, as shown in Figure 4g. The comparison between the harmonic intensity and the fundamental part of the power amplifier shows that the quality of nonlinearity could be improved by decreasing the duty cycle. Moreover, the harmonic intensity is greater than the fundamental when the duty cycle is less than 0.2. For the case when the duty cycle is 0.5% or 50%, from Figure 4g, we observe that the gain of the first positive harmonic of the power amplifier is positive, implying that the amplifier could amplify the fundamental wave and the first positive harmonics wave simultaneously. Figure 4f implies that the first odd harmonic components are depressed within 5 dB compared with the fundamental part, indicating that the power amplifier has strong nonlinearity.

#### 2.2.4 The Proposed APTSTCM Element

**Figure 5:**
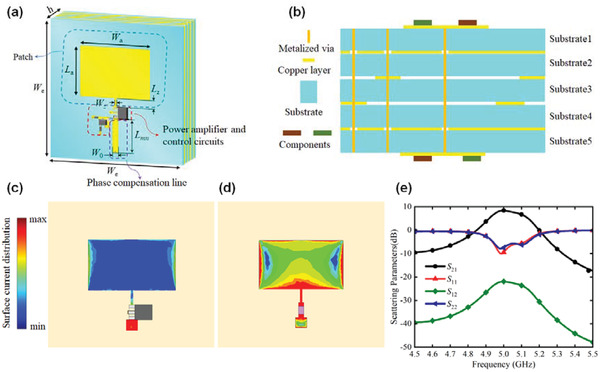
APTSTCM unit cell, which could enhance the incident wave. a) 3D view of the APTSTCM element. b) Side view of designed space‐time coding metasurface. c,d) Full‐wave simulated electric intensity distributed on the receiving and transmitting patch, respectively. e) Simulation results of scattering parameters for APTSTCM element.

In order to minimize the structure of the APTSTCM element and integrate the electronic components with metasurface, the proposed APTSTCM element is fabricated in the multilayer PCB, as depicted in **Figure** **5**a,b. The phase compensation line is placed on both sides of the metasurface, connected by a metal hole through the metasurface. When an incident wave impinges the APTSTCM element, the amplifier could enhance the signal from the receiving patch and transmit it via phase compensation line to the transmissive patch, and then radiate it to the space in the desired manner.

The top and bottom layers are placed with the patches and microwave circuits. The middle layers are ground planes except for the third and fourth layers (one is for the bias control signal, and the other is for the DC power supply). Additionally, ground planes between receiving patch and transmissive patch improve the isolation of the APTSTCM element. The relative dielectric constants of each substrate of APTSTCM element are *ε*_1_ = *ε*_5_ = 3.7, *ε*_2_ = *ε*_3_ = *ε*_4_ = 4.4. And the thickness of each substrate is *h*_1_ = *h*_5_ = 1.524 mm, *h*_2_ = *h*_4_ = 0.096 mm, *h*_3_ = 0.08 mm. For predicting the performance of the APTSTCM element, field and circuit co‐simulation were performed by HFSS and Circuit simulator in ANSYS Electronics Desktop 2018. And the measured small‐signal scattering parameters of the amplifier were applied as the circuit module. From Figure 5c,d, the simulated surface current distribution on the patch intuitively verifies the electric intensity enhancement of the APTSTCM element. Figure 5e is the co‐simulated scattering parameters results of the APTSTCM element, which indicates the enhancement and nonreciprocity of the APTSTCM element. Observe that more than 30 dB isolation can be achieved to ensure the stability of the APTSTCM element and metasurface. It must be pointed out that the above‐simulated results are all based on the unilateral amplifiers, which are switched to ON status controlled by the bias control circuit. Furthermore, the harmonics generation could be achieved by periodically modulating the amplifier via the bias control circuit. With the adjustment of the space‐time coding matrix, arbitrary phase and magnitude of harmonics could be reached for reshaping the radiation pattern in the space.

### 2.3 Experimental Verification

**Figure 6:**
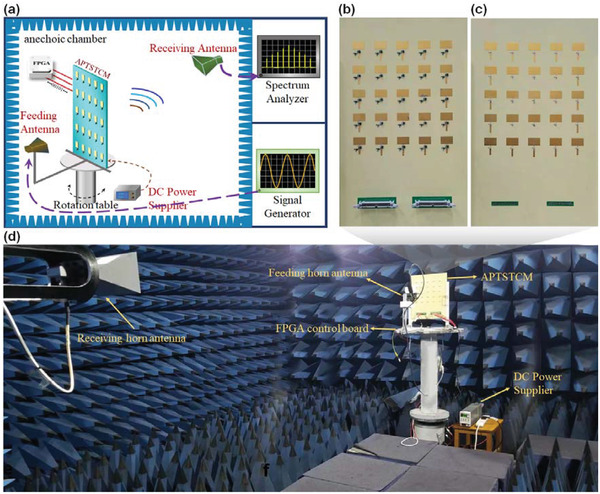
Measurement setup. a) Schematic of the measurement setup. b,c) Top and bottom view of fabricated space‐time coding metasurface prototype. d) Photograph of the experimental environment in a microwave anechoic chamber.

The schematic of the measurement setup is depicted in **Figure** **6**a. The prototype and feeding horn antenna are fixed on the rotation table. For exciting the feeding horn antenna, vector network analyzer N5230A of Keysight is applied as a signal generator. Additionally, for receiving the harmonic power intensity radiated from the APTSTCM prototype, the receiving horn antenna, a pyramidal horn antenna, is connected with a spectrum analyzer (Agilent N9918A). In order to verify the concept of APTSTCM, a 1‐bit APTSTCM prototype consisting of 5 × 5 APTSTCM elements is fabricated with dimensions 400 mm × 280 mm × 3.484 mm, as shown in Figure 6b,c. The APTSTCM prototype has 50 control lines for the DC power supply to amplifiers and the bias control circuit. Half of the lines are digitally control lines connected with each I/O port of FPGA via a 40 pin ribbon cable connector shown at the bottom‐right of Figure 6b. The rest are DC power lines to amplifiers connected with the DC power supplier via another 40 pin ribbon cable connector. Therefore, each APTSTCM element can be directly controlled by FPGA. Besides that, flexible bias voltage adjustment of amplifiers can be realized by tuning the output voltage of the DC power supplier.

**Figure 7:**
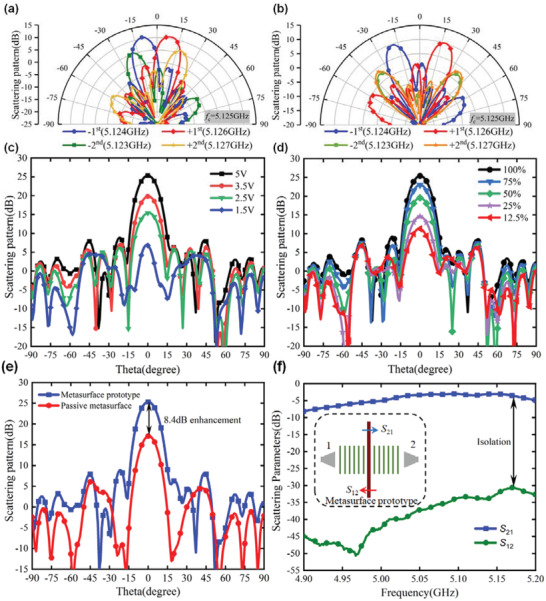
Experimental results. a,b) Measured scattering patterns at harmonic frequencies, pointing at lower and larger scanning angles, respectively. c,d) Measured scattering patterns at different voltages of biasing voltage to amplifiers and different proportions of code “1” and “0” in the time coding sequence at frequency 5.125 GHz, respectively. e) Comparison of passive metasurface and 1‐bit APTSTCM far‐field scattering patterns and gain at frequency 5.125 GHz. f) Measured scattering parameters of APTSTCM prototype for the strong nonreciprocity.

The 1‐bit APTSTCM was fed through a horn antenna located 280 mm above it. The feeding antenna is a broadband ridge horn antenna with an aperture size of 77.9 mm × 103.8 mm. For achieving the accurate power distribution of harmonics of APTSTCM in the far‐field, the metasurface prototype is measured in a microwave anechoic chamber. An FPGA control board is utilized to yield dynamic digital voltage for the APTSTCM. Figure 6d shows the photograph of the actual experimental environment in the microwave anechoic chamber. **Figure** **7**a,b shows the measured horizontal scattering pattern at an elevation of 0°, corresponding to the first two positive and negative harmonics at the central frequency of 5.125 GHz. The harmonic beams scanning at wider angles are achieved with different code sequences in the space‐time coding matrix. For the harmonic generation, the modulation frequency is 1 MHz, which corresponds to the modulation period of the space‐time coding matrix is 1 us, and pulse width *σ* is 0.25 us. Based on the synthesized method Equation (6), the phase of the *p*th harmonic outgoing wave (2*l* − 1)/2*pω*_0_ *σ* of the unit cell on the metasurface depends on the time sequence code, corresponding to the binary code in the space‐time coding matrix. The magnitude of the *p*th harmonic is mainly determined by the coefficients *sin* *c*(*pω*_0_ *σ*/2), which is a constant when the proportion of code “1” in the time sequence is fixed. Then the phase and magnitude of the *p*th harmonic could be adjusted independently to manipulate the intensity and steering angle of the outgoing harmonic wave separately. From a theoretical aspect, the resolution *p*π/*L* in phase manipulation could be accurate enough to examine the experimental results, and the radiation patterns of positive and negative harmonics are symmetrical in terms of the symbol *p* in Equation (6). We also note that the harmonic steering angle increases with the harmonic order. The *p*th harmonic steering angle fulfills the relation *θ_p_* = arcsin(*p*sin(*θ*_1_)), where *θ*_1_ is the steering angle of the first positive harmonic wave.

The binary code of the space‐time coding matrix corresponds to the square wave in the time domain, controlling the status of the power amplifier integrated with each unit cell of the metasurface. We can manipulate the scanning angles of the harmonic waves by adjusting the position of the square wave in periodic time. Different positions of the controlling square wave in the time sequence correspond to the distinct phases of the outgoing harmonic waves on each unit cell of the metasurface. The time sequence of the left bottom unit cell on the metasurface is regarded as a time integral reference to calculate the phase distribution of harmonics. Due to the Fourier transform of the square wave, all phases and magnitudes of harmonic components are generated simultaneously. From the experimental aspect, the minimum lasting time of the square wave output from the FPGA board applied to switch the power amplifier integrated on the metasurface is 20 ns, which is high resolution to the modulation period 1 us, determining the length of time sequence (*L =* 1 us/20 ns) and the phase distinction *p*π/*L* of the *p*th harmonic wave in the experimental setup. The *M*×*N*×*L* dimension space‐time coding matrix is used to generate and manipulate the harmonics waves, where *M* and *N* depend on the numbers of unit cells on the vertical and horizontal direction of the manufactured metasurface, as depicted in Figure 6b,c, which is 5 by 5. Two different cases are evaluated for verifying the proposed method. From Figure 7a, the first positive harmonic steering angle 9.94° is calculated to achieve the phase distribution, which is quantized in *M*×*N*×*L* space‐time coding matrix and stored in the FPGA board. Other harmonic steering angles are generated simultaneously based on Equation (6). In another case, a larger steering angle of the first positive harmonic is designed as 19.47°, as shown in Figure 7b. The fundamental harmonic radiation patterns with different duty cycles are measured and presented in Figure 7d, implying that the intensities of all harmonics could be adjusted by tuning the code “1” proportion in the binary code of the metasurface.

Considering that the periodic distribution of each unit cell on the manufactured metasurface prototype is 3*λ*_c_/4 (45 mm), where *λ*_c_ is the wavelength of the incident central frequency 5 GHz in the free space, the wider outgoing harmonic waves have a much larger grating lobe. For the measured results shown in Figure 7b, the grating lobe is almost symmetrical to the main beam of the harmonic wave when the steering angles of the ±2nd harmonic waves are ±41.81°. The grating lobe of the +2nd harmonic wave almost overlaps to the main beam of the −2nd harmonic wave and vice versa. The comparison of steering angular of harmonic beams between theoretical calculation and measured results is listed in **Table** **1**, indicating that experimental results highly agree with the theoretical prediction. Furthermore, there are two methods of amplification control to fundamental harmonic beam based on the theory of transmission coefficient. One is adjusting the output voltage of the DC power supplier to coding metasurface, as shown in Figure 7c. Another is changing the proportion of codes “1” and “0” in the space‐time coding sequence shown in Figure 7d. Based on Equation (8), the gain of the power amplifier is determined by the conduction angle *ϕ*, which is related to the DC bias voltage to the power amplifier. As the DC voltage on the power amplifier increases, the conduction angle becomes larger, leading to the gain enhancement of the power amplifier, which improves the intensity of the fundamental wave, as depicted in Figure 7c.

**Table 1:** Comparison between the theoretical calculation and measured results

Beam steering at different harmonic frequencies	−2nd [degree]	−1st [degree]	+1st [degree]	+2nd [degree]
Theoretically calculated steering angle	−20.18	−9.94	+9.94	+20.18
Measured steering angle	−18	−10.55	+10.63	+20
Theoretically calculated steering angle	−41.81	−19.47	+19.47	+41.81
Measured steering angle	−40	−20	+18	+38

By comparing the radiation pattern of the passive surface and the fundamental harmonic beam of 1‐bit APTSTCM, more than 8 dB power enhancement is achieved when each element of the space‐time coding matrix is full of code “1,” as shown in Figure 7e. We use the standard horn antenna, the feeding antenna of APTSTCM, as a reference for obtaining the absolute gain, with a known gain 11.7 dBi. By recording the power intensity of harmonic frequencies in the azimuth direction at an elevation of 0°, compared with standard horn antenna, the absolute gain of APTSTCM is achieved under different modulation methods. Figure 7f shows excellent isolation of the APTSTCM prototype with almost over 25 dB. In other words, measured scattering parameters imply the strong nonreciprocity of APTSTCM. Compared with the APTSTCM design, a 125 MHz center frequency shift was observed because of the fabrication process's tolerance, the components difference, the gap between the scattering parameter model of components, and the coupling effect of adjacent elements on the metasurface. In summary, the measured results verify the experiment's validity and give solid support to our proposed approach and method of APTSTCM synthesis.

## 3 Conclusion

We presented a new amplifier‐based transmissive space‐time coding metasurface by using the strong nonlinearity of the power amplifier to enhance the power intensities at harmonic frequencies. Unlike the other lossy components applied on the space‐time coding metasurface, APTSTCM has the capabilities of harmonic engineering and can enlarge the operating distance of the transmitted EM wave. A 1‐bit APTSTCM was fabricated and measured in terms of the synthesis of APTSTCM. The experimental results are in good agreement with the theoretical predictions of the 1‐bit APTSTCM. Although the working bandwidth of the 1‐bit APTSTCM prototype is narrow, it can be extended to broadband devices by increasing the operating bandwidth of the patch and power amplifier. Moreover, the higher gain of APTSTCM could be guaranteed by using a high‐gain power amplifier to improve the signal quality further. By dynamic periodic switching to the power amplifier according to the space‐time coding matrix, the harmonic beams could be generated and radiated in space with the desired scattering patterns, which has great importance in wireless communications and energy transmissions. Furthermore, the nonreciprocity of the unilateral power amplifier makes APTSTCM have the capabilities to unilaterally transmit the EM wave. By encoding APTSTCM via changing the proportions of “0” and “1” in the time coding sequence, we have achieved the digitally adjusted harmonic frequency power distributions in the far‐field space with predefined patterns. Additionally, APTSTCM could realize broader coverage and higher signal quality, which is of considerable practical significance in radar systems and wireless repeaters.

## Conflict of Interest

The authors declare no conflict of interest.

## Data Availability

The data that support the findings of this study are available on request from the corresponding author. The data are not publicly available due to privacy or ethical restrictions.
